# Improvement of myocardial perfusion reserve detected by cardiovascular magnetic resonance after direct endomyocardial implantation of autologous bone marrow cells in patients with severe coronary artery disease

**DOI:** 10.1186/1532-429X-12-6

**Published:** 2010-01-25

**Authors:** Carmen Wing-Sze Chan, Yok-Lam Kwong, Raymond Y Kwong, Chu-Pak Lau, Hung-Fat Tse

**Affiliations:** 1 Cardiology Division, Department of Medicine, University of Hong Kong, Queen Mary Hospital, Hong Kong; 2 Haematology Division, Department of Medicine, the University of Hong Kong, Queen Mary Hospital, Hong Kong; 3 Cardiac Magnetic Resonance Imaging, Cardiovascular Division, Brigham and Women's Hospital, Harvard Medical School, Boston, USA

## Abstract

**Background:**

Recent studies suggested that bone marrow (BM) cell implantation in patients with severe chronic coronary artery disease (CAD) resulted in modest improvement in symptoms and cardiac function. This study sought to investigate the functional changes that occur within the chronic human ischaemic myocardium after direct endomyocardial BM cells implantation by cardiovascular magnetic resonance (CMR).

**Methods and Results:**

We compared the interval changes of left ventricular ejection fraction (LVEF), myocardial perfusion reserve and the extent of myocardial scar by using late gadolinium enhancement CMR in 12 patients with severe CAD. CMR was performed at baseline and at 6 months after catheter-based direct endomyocardial autologous BM cell (n = 12) injection to viable ischaemic myocardium as guided by electromechanical mapping. In patients randomized to receive BM cell injection, there was significant decrease in percentage area of peri-infarct regions (-23.6%, *P *= *0.04*) and increase in global LVEF (+9.0%, *P *= *0.02*), the percentage of regional wall thickening (+13.1%, *P= 0.04*) and MPR (+0.25%, *P *= *0.03*) over the target area at 6-months compared with baseline.

**Conclusions:**

Direct endomyocardial implantation of autologous BM cells significantly improved global LVEF, regional wall thickening and myocardial perfusion reserve, and reduced percentage area of peri-infarct regions in patients with severe CAD.

## Background

Recent clinical studies [1-5] suggest that direct implantation of autologous bone marrow (BM) cells into the ischaemic myocardium improves symptoms and exercise capacity and increases left ventricular (LV) function in patients with severe coronary artery disease (CAD). Despite the absence of trans-differentiation of transplanted BM cells into ischaemic myocardium, improvement in LV function is observed in experimental studies [6,7]. Indeed, emerging evidences from experimental studies indicate than BM cells might exert their benefit via paracrine effects to induce angiogenesis in myocardial ischaemia [6,8,9]. However, there is very limited data on the functional effects of direct BM cells implantation in human [1,2]. Advances in cardiovascular magnetic resonance (CMR) allow non-invasive assessment and serial monitoring of myocardial perfusion by capturing the first-pass perfusion of a bolus of gadolinium injection [10-13]. Furthermore, the use of late gadolinium enhancement (LGE) CMR can identify and quantity the amount of heterogeneous zone of viable and nonviable peri-infarct myocardium, which has been shown to be a powerful predictor for mortality in post myocardial infarction patients [14]. In this study, we sought to investigate the changes in myocardial function, perfusion and myocardial scar using CMR imaging in patients with severe CAD following direct endomyocardial BM cells implantation.

## Methods

### Study Population

Among those 28 patients enrolled into a Phase I-II randomized PROTECT-CAD trial to evaluate the safety and feasibility of using a catheter-based intramyocardial autologous BM cells implantation [15], we performed a subgroup analysis in 12 patients recruited in Hong Kong who had received BM cells injection in whom detailed CMR was performed using the same imaging protocol. The details of inclusion, exclusion criteria and study protocol have been reported previously [15]. As reported previously [15], patients treated with BM cell injection had significant improvement in global and regional LV function as measured by CMR compared with controls. This is a post-hoc exploratory analysis to study the changes in CMR parameters in patients treated with BM injections which were not included in the predefined analysis of the PROTECT-CAD trial.

### Study Protocol

In brief, all patients enrolled into PROTECT-CAD trial were suffered from severe CAD with reversible perfusion defects detected by single-photon emission computed tomography and had no other option for conventional revascularization therapies. All patients gave informed consent, and the protocol was approved by the institutional review boards. The study population was randomized to receive direct intramyocardial injection of either BM cells (BM group) or autologous plasma (controls) into chronic ischemic myocardium as guided by electromechanical mapping in 2:1 ratio [15].

In this sub-study, all patients were randomized to the BM group. As reported previously [1,15], mononuclear cells at a concentration of 1 × 10^7 ^cells per ml were prepared by Ficoll density gradient centrifugation of autologous BM cells harvested from the right iliac crest in each patient. The cell viability was >95% and intramyocardial injection was performed immediately after preparation (~3 hours after harvesting). All patients underwent non-fluoroscopic LV electromechanical mapping (NOGA system, Biosense-Webster) to identify targeted ischaemic myocardium which was matched SPECT and CMR perfusion imaging [1-5,15]. At each targeted ischaemic region, injections of 0.1 ml of mononuclear cell suspension were delivered as guided by electromechanical mapping [15].

### CMR Protocol

#### Cine CMR

CMR was performed at baseline and at 6 months after catheter-based direct endomyocardial autologous BM cell, and within one week after the single-photon emission computed tomography examination. All patients were examined in the supine position with a 1.5 T system (CV/I, General Electric, Milwaukee, Wisconsin), using a four-element phased-array cardiac coil. All images were obtained during repeated breath-holds. Cine images were performed in multiple parallel short-axis planes covering the entire LV using an ECG-gated segmented K space, steady state free precession pulse sequence as previously reported [14-16].

#### Adenosine First Pass Perfusion CMR

An intravenous bolus of 0.05 mmol/kg gadolinium-DTPA (Magnevist, Schering) was administered at a rate of 5 ml/s by a power injector 4 minutes after continuous adenosine infusion at 140 mg/kg/min. All patients were under continuous blood pressure, pulse oximetry and electrocardiogram monitoring. First-pass perfusion imaging was performed simultaneously with contrast injection for the first 30 to 60 heart beats, using a saturation recovery interleaved fast gradient echo-echo planar pulse sequence at three short-axis left ventricular level (basal, mid ventricular and apical) as previously reported [14-16]. After a twenty minute waiting period for equilibration of the contrast agent within the myocardium, the resting first pass perfusion imaging using the same imaging parameters and same slice locations were acquired.

#### Late Gadolinium Enhancement (LGE) CMR

According to a previously described inversion recovery pulse sequence (repetition time, 4.8 ms; echo time, 1.4 ms; in-plane spatial resolution between 1.5-1.8 mm and 1.8-2.1 mm)[17], LGE images at matching cine-image slice locations were required 10 to 15 minutes after cumulative dose of 0.15 mmol/Kg intravenous gadolinium-DTPA administration. The inversion time (250 to 300 ms) was optimized to null the normal myocardium and adjusted the views per segment and trigger delay according to the patient's heart rate.

### CMR Image Analysis

All images were reviewed and analyzed off-line with specialized post-processing software (Cinetool version 3.9.8, General Electric Healthcare) by two investigators (WSC and RYK). Analysis and interpretation of all imaging data were blinded to the clinical data and outcome. The LV volume measurement, calculation of ejection fraction and regional wall thickening were analyzed using the same technique which was reported in the previous study [18,16]. The LV volumes were determined by planimetry at the end-systolic and end-diastolic frame, and the LV myocardial mass was calculated by subtracting the endocardial volume from the epicardial volume at end diastole and then multiplying by the tissue density (1.05 g/ml).

### First-Pass Perfusion Analysis

All first-pass perfusion images were analyzed in short-axis planes according to the standardized myocardial segmentation and nomenclature for tomographic imaging of the heart [19], with each equiangular segment per slice assigned to a coronary artery territory. For perfusion analysis, the endocardial and epicardial contours were manually traced and corrected for displacement during breathing. The myocardial signal intensity and arterial input function measured in the basal LV slice were determined for all time points. The myocardial blood flow was determined by deconvolution of myocardial signal intensity curves with an arterial input function measured in the LV blood pool by using the Fermi model and expressed in mL/min/gm [20,21]. The myocardial reserve was calculated from the ratio of myocardial blood flow during adenosine infusion to myocardial blood flow at rest and used as an index for the detection of any changes of myocardial perfusion after BM injections.

### Infarct and Peri-infarct Size Analysis

**Figure 1:**
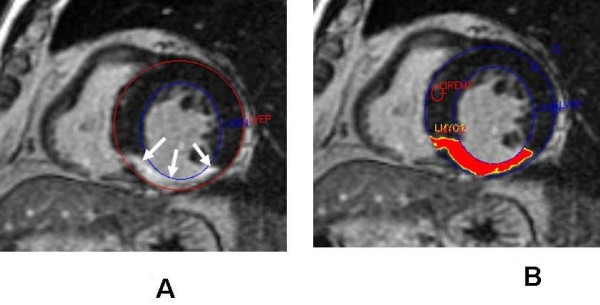
**(A) The endocardial (LVEN) and epicardial (LVEP) borders at the end diastole of delay enhancement image were trace manually**. The white arrows indicating the extension of transmural myocardial infarction shown up as area of LGE. (B) A computer-assisted semiautomatic technique was applied to quantify the %MDE_*peri-infarct*_. The computer algorithm then used the signal-intensity thresholds of >3 SDs and 2 to 3 SDs above the normal myocardial segment (REMT) to delineate the infarct core (LYMO1- red region) and peri-infarct area (LYMO 2-yellow region) respectively.

The endocardial and epicardial contours on delayed enhancement images were traced manually (Figure 1). By using a semiautomatic detection algorithm, a signal-intensity threshold of 2 standard deviations (SDs) above a reference remote myocardial region on the same slice was applied to quantify the total infarct mass (MDE_*total*_) [10,22,23]. It was further partitioned into the strongly enhanced core infarct mass (MDE_*core*_) and the peri-infarct mass (MDE_*peri*-*infarct*_) based on signal-intensity thresholds of 3 SDs and 2 to 3 SDs above the remote reference segment, respectively. Infarct size (%MDE_*total*_) was expressed as a percentage of LV mass. Furthermore, normalized MDE_*peri*-*infarct *_and MDE_*core *_as percentages of the MDE_*total *_were calculated as previously described [14].

### Statistical Analysis

Data are reported as means ± 1 standard derivation. Comparisons between paired variables were performed using Student's t test, with a significance level of P < 0.05. All statistical analyses were conducted with SPSS version 13.

## Results

The baseline characteristics of the study population are shown in Table 1. A total of 190 percutaneous catheter-based endomyocardial injections to 21 ischaemic regions (inferior = 6, lateral = 5, septal = 5, and anterior = 5) as guided by the electromechanical mapping were performed in 12 patients without any acute complication. On average, 16 ± 4 (range 9-23) injections per patient were performed.

**Table 1:** Baseline characteristics

	N = 12
**Age, years**	66 ± 9
**Men, n (%)**	9 (75)
**Diabetes mellitus, n (%)**	7 (58)
**Hyperlipidaemia, n (%)**	12 (100)
**Hypertension, n (%)**	10 (83)
**Current cigarette smoker, n (%)**	6 (50)
**Percutaneous coronary intervention, n (%)**	10 (83)
**Coronary artery bypass surgery, n (%)**	7 (58)
**Injected treatment site, n (%):**	
Inferior wall	7 (32)
Lateral wall	2 (9)
Anterior wall	5 (23)
Septal wall	8 (36)
**Medication at baseline, n (%)**	
Angiotensin-converting enzyme inhibitors or angiotensin-receptor blockers	6 (50)
Aspirin and/or clopidogrel	16 (100)
β- blockers	12 (100)
Calcium channel blockers	8 (67)
Nitrates	12 (100)
Statins	16 (100)
**Medication at 6 months, n (%)**	
Angiotensin-converting enzyme inhibitors or angiotensin-receptor blockers	6 (50)
Aspirin and/or clopidogrel	16 (100)
β- blockers	12 (100)
Calcium channel blockers	7 (58)
Nitrates	11 (92)
Statins	16 (100)

### CMR Analysis

There was no significant difference in terms of the haemodynamic conditions of patients at 6 months compared with baseline. Compared with baseline, there were significant improvement in LV ejection fraction (51 ± 10% versus 56 ± 9%, *P *= *0.02*) and regional LV wall thickening over the targeted regions (48.1 ± 10.2% versus 53.4 ± 8.6%, *P *= *0.03*) at 6-month. However, there were no significant changes in the LV end diastolic volume (150 ± 33 ml versus 146 ± 30 ml, *P *= *0.70*) and LV mass (99 ± 28 gm/m^2 ^versus 95.6 ± 28.5 gm/m^2^, *P *= *0.75*) between 6-month and baseline.

### Changes in Myocardial Perfusion Reserve

Table 2 shows the values of myocardial blood flow at rest and during adenosine stress at the targeted and non-targeted region at baseline and 6 month follow-up. At baseline, the myocardial perfusion reserve in all patients was significantly lower at the targeted regions as compared to non-targeted regions (1.2 ± 0.2 versus 2.1 ± 0.6, *P *= *0.002*), suggestive of significant myocardial ischaemia at the targeted regions.

**Table 2:** Myocardial blood flow at rest and during adenosine stress at the targeted and non-targeted region at baseline and at 6 months after bone marrow cell injection

	Baseline		6 month follow-up	
	
	Rest mL/min/gm	Adenosine Stress mL/min/gm	Rest mL/min/gm	Adenosine Stress mL/min/gm
**Targeted regions**	0.67 ± 0.17	0.83 ± 0.16	0.75 ± 0.3	1.12 ± 0.5
**Non-targeted regions**	0.97 ± 0.9	2.01 ± 0.7	1.01 ± 0.5	2.3 ± 0.7

**Figure 2:**
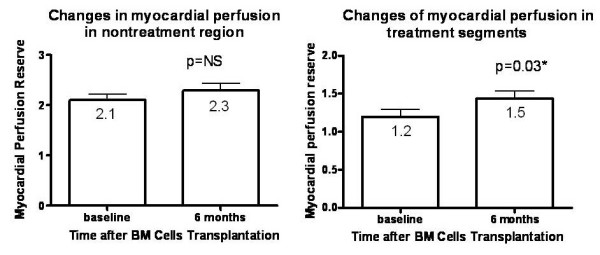
**Treatment effect of bone marrow (BM) cells implantation on myocardial perfusion reserve in targeted and non-targeted regions in the BM group as determined by CMR**. Data presented as mean ± SD (error bar).

As shown in Figure 2, the myocardial perfusion reserve at the targeted regions after BM cell injection was significantly increased at 6-month compared with baseline (1.2 ± 0.3 versus 1.5 ± 0.4, *P *= *0.03*). Nevertheless, there was no change in myocardial perfusion reserve at the non-targeted regions between 6-month and baseline (2.1 ± 0.6 versus 2.3 ± 0.6, *P *= *0.20*).

### Tissue Characterization by LGE

**Figure 3:**
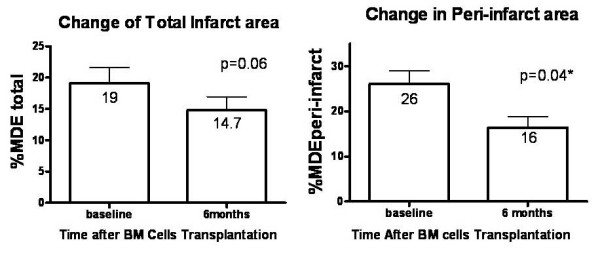
**Treatment effect of bone marrow cells (BM) implantation on percentage of total infarct area and peri-infarct area in the BM group as determined by CMR**. Data presented as mean ± SD (error bar).

LGE was present in all patients, consistent with prior myocardial infarction. 75% patients had <50% transmural infarction while the remaining patients had 50-75% transmural infarction. Compared with baseline, there was an insignificant modest reduction in the %MDE_*total *_at 6-month (-23%, *P *= *0.06*) after BM cell injection. Nevertheless, there was a significant decrease in %MDE_*peri*-*infarct *_at 6-month as compared with baseline (-38%, *P *= *0.04*, Figure 3). However, there was no significant difference in the transmural extension of LGE (*P *= *0.8*)

## Discussion

The results of this study demonstrate that direct intramyocardial implantation of BM cells was associated with increased myocardial perfusion reserve and regional LV wall thickening at the targeted ischemic regions. Furthermore, there was a reduction in the extent of ischemic peri-infarct area detected by CMR, without any significant decrease in the extent of core infarct area after BM cells injection. Taken together, these CMR findings suggest that BM cells implantation improve myocardial perfusion reserve and reduce ischaemic peri-infarct region in human ischemic myocardium, which subsequently contribute to an improvement in regional LV function.

In this study, the mean myocardial perfusion reserve in the targeted ischaemic regions was significantly lower than the non-targeted regions, indicating that the targeted regions have more tissue ischaemia at baseline. The improvement in myocardial perfusion reserve was related to an increase in peak flow rather than a decrease in baseline flow, suggesting enhancement of stress induced myocardial blood flow after BM cell implantation. This postulation is further evidenced by the reduction in the extent of ischaemic myocardium at 6-month after BM cells implantation compared with baseline.

Furthermore, the lack of significant reduction in core infarct area after BM cells implantation indicates the lack of significant myocardial regeneration over the scar area. These results are consistent with the findings in recent experimental studies which showed limited capability of BM cells to trans-differentiate into cardiomyocytes and continued to differentiate along the hematopoietic lineage after transplantation [5,6,24]. However, an improvement in LV function was consistently observed in small [5,6] and large animal studies [7,8,25,26] after BM cells transplantation. Experimental studies have suggested that the majority of the benefit effect of BM cells implantation might exert via paracrine mechanisms with secretion of various angiogenic cytokines, such as vascular endothelial growth factor to induce angiogenesis in myocardial ischemia [8,25,26]. Nevertheless, it remains unclear whether similar paracrine mechanisms contribute to the improvement in myocardial perfusion reserve as observed in the present study.

### Study Limitations

First, a small sample size of this study is a limitation as only patients with detailed CMR examination using the same protocol and machine were included in this analysis. Therefore, our results need to be confirmed by larger, prospective, randomized studies. Second, although the cutoff of remote +2 SD for the entire infarct has been validated in previous studies and currently being practicing in daily clinical application, future studies are still needed to validate the used a pre-specified, arbitrary signal intensity threshold (remote+ 3 SD) above the remote myocardium to delineate the core infarct on LGE. Final, whether the treatment effects of BM cells implantation noted after 6 months are sustained over time needs to be studied.

## Conclusion

In conclusion, our data from CMR provide insights into the potential functional effects of catheter-based direct intramyocardial delivery of BM cells in the chronic human ischemic myocardium.

## Competing interests

The authors declare that they have no competing interests.

## Authors' contributions

WSC and RYK were responsible for CMR study design, reading, data analysis and manuscript preparation. HFT, YLK and CPL were responsible study design, patients recruitment and procedure, and manuscript preparation.
